# pH regulated lactose inspired fabrication of zinc oxide nanoparticles for insulin sensing by LSPR absorption^☆^

**DOI:** 10.1016/j.heliyon.2023.e18153

**Published:** 2023-07-16

**Authors:** Nasim Ahmed, Shaikat Chandra Dey, Nusrat Mustary, Md Ashaduzzaman

**Affiliations:** a Department of Applied Chemistry and Chemical Engineering, Faculty of Engineering and Technology, University of Dhaka, Dhaka, 1000, Bangladesh; b Institute of Fuel Research and Development (IFRD), Bangladesh Council of Scientific and Industrial Research (BCSIR), Dr. Qudrat-I-Khuda Road, Dhaka, 1205, Bangladesh; c Department of Forest Biomaterials, North Carolina State University, Campus Box 8005, Raleigh, NC, 27695, USA; d Department of Community Medicine, Dhaka National Medical College, 53/1 Johnson Road, Dhaka, 1100, Bangladesh; e Institute of Advanced Materials, Ulrika, Linkoping, Sweden

**Keywords:** pH regulated, LSPR, ZnO nanoparticle, Lactose, Insulin

## Abstract

Nanostructured metal oxide particles with diversified morphologies are in high demand in nanotechnology. The particle size, shape, and overall geometry mainly depend on the fabrication method. This study reports synthesis of zinc oxide nanoparticles (ZnO NPs) from zinc nitrate hexahydrate [Zn(NO_3_)_2_.6H_2_O] precursor in aqueous media at 65 °C by using lactose from cow milk as a reducing agent and regulating pH from 6 to 10. UV–visible absorption gave maximum absorbance (*λ*_max_) at 371–375 nm in ethanol for localized surface plasmon resonance (LSPR), FTIR exhibited bands at ca. 439–481 cm^−1^ for stretching mode Zn–O bonds, and XRD peaks at 2 θ values at 31.8, 34.45, and 36.28° confirmed the fabricated ZnO NPs. The XRD spectra also indicated that the ZnO crystallite (20–30 nm) has a hexagonal wurtzite structure. The average particle sizes measured by DLS were ca. 50–837 nm, and SEM microphotographs demonstrated the morphology of ZnO NPs with a hexagonal, rod-shaped, or spike-like structure. The ZnO NPs were used to investigate the LSPR absorption at various concentrations of insulin, ranging from 2.5 μM to 50 μM. The ZnO NPs fabricated at pH 7 and 10 showed better insulin sensing performance with high precision. The synthesis approach of ZnO NPs with variable morphologies would play a significant function in biomedical science especially real time monitoring of glucose for efficient management of diabetes.

## 1 Introduction

Nowadays, nanostructured materials have earned tremendous attraction due to their size, structure, shape, and overall morphologies suitable for pragmatical applications in the areas of nanobiotechnology, nanomedicine, nanobioelectronics, etc. [[1], [2], [3], [4], [5], [6], [7]]. On the basis of nanomaterials, innovation in modern nanotechnology for biomedical treatment is the new hope for mankind in the future [1]. Physical and chemical behaviors of inorganic nanomaterials vary with size and shape due to their electronic existence on the surface and their enhanced reactivity. Green technologies with regulating nanostructure syntheses are still challenging for nanobiotechnology [[3], [4], [5]]. Interaction between nanoparticles and biomolecules could create detection technology for sensing, bioimaging, cancer diagnosis and target drug delivery [8].

Considering the target of applications, the methodologies of nanoparticle syntheses would have a vital role because the synthesized nanoparticles bear reacting elements of the system. Naturally occurring biomolecules would help reduce the problems arising from other methods. Various molecules such as alkaloids, terpenoids, flavonoids, amino acids, enzymes, vitamins, proteins, and glycosides extracted from plants are widely used for the green synthesis of different nanostructures [[9], [10], [11], [12], [13], [14], [15]]. Besides, lactose, a large-scale source of biomolecules available after skimming proteins from cow milk, offers the potential for the production of nanomaterials by complexation with cations for bioreduction, nucleation, formation, and stabilization.

Morphologies of ZnO nanoparticles depend mainly on the sources of precursors used for fabrication [14]. In our previous article, we reported saponin-imprinted ZnO nanohoneycomb synthesis from ZnSO_4_ and its toxic responses against selective bacteria [2]. We also reported a green microwave-assisted fabrication of spherical ZnO nanoparticles and applications for photocatalysis against a dye as well as for antibacterial activity [3]. Qiang Huang and Jianping Liu reported facile preparation of ZnO nanorods assisted with Aliquat 336 from Zn(CH_3_COO)_2_ [16]. Getie et al. reported the fabrication of nanorods and spherical ZnO nanoparticles from different zinc precursors such as Zn(NO_3_)_2_, ZnCl_2_, and Zn(CH_3_COO)_2_ [17]. The value of pH has a significant impact on the characteristics of ZnO when it is synthesized by sol-gel technique. During gel formation, the pH of the solution directly influences the hydrolysis and condensation behaviors, and consequently the morphology of the ZnO [18]. For example, Li et al. demonstrated that the solution conditions have a specific influence on the particle size of ZnO powder [19]. Additionally, the pH can affect the number of ZnO nuclei and growth units [20]. According to Sagar et al., the increase in pH (from acidic to alkaline) of the sols caused the formation of a ZnO film [21]. In this article, we have reported for different structures of ZnO nanoparticle synthesis from a single source, Zn(NO_3_)_2_, only by regulating the pH of the cow milk solution, which governs the pattern of ZnO nanoparticle morphologies. By regulating pH, it is possible to control the ZnO nanoparticle yield, crystallinity, particle size, morphology, which ultimately effect the LSPR absorption. The change in LSPR can be utilized to sense important biomolecule such as insulin [5,22].

Nanostructured ZnO particles are interesting among the metal oxide nanoparticles due to their wide band gap, large binding energy, and high piezoelectric characteristic [23]. ZnO NPs have already proven to be biosafe, nontoxic, and biocompatible and are used in various industrial and technological fields, including electronics, sensors, diagnosis, medicine, processing, environmental pollution cleansing, etc. [[24], [25], [26], [27], [28], [29]].

Varsha Thambi et al. reported the preparation of pH-controlled overgrowth of gold nanorod complex nanoparticles with different geometries [30], and Xing Zhang et al. also reported the fabrication of pH-regulated monodisperse penta-twinned gold nanoparticles, e.g., spheroid, bipyramid, and decahedron, with high yield. It was discovered that the effect of pH was primarily attributable to the regulation of the reduction potential of reductants (RPRs). This regulates the atomic deposition preference on the penta-twinned seed surface due to the lattice strain energy at the twin boundary, which then determines how it grows into final products [31].

LSPR is an intrinsic behavior of metal nanostructure that has been previously reported by different research groups [[32], [33], [34], [35]]. The local electromagnetic characteristic is remarkably magnified by many optical phenomena such as absorbing, scattering, transmission, etc. [[36], [37], [38], [39]]. In 2008, Kreuzer et al. quantitatively determined the synthetic anabolic steroid called stanozolol on gold-modified glass substrates primarily functionalized with biotin and measured by conventional dark-field spectroscopy [39].

The interaction of ZnO nanoparticles with biological molecules is one of the most significant and difficult issues in molecular biology. Molecular dynamics (MD) models are a good way to figure out how proteins and nanoparticles interact with each other. The interaction mechanism of insulin with ZnO nanoparticles was investigated using MD and replica exchange molecular dynamics (REMD) simulation techniques, as well as their conditions. It was understood through the use of REMD modeling that insulin interacts with the surface of ZnO nanoparticles in a manner that is mediated by the polar and charged amino acids that it possesses, when insulin is allowed to unfold on the surface of a ZnO nanoparticle, the most important parts of its chains are the ones at the ends [8].

For the first time here, we have reported the sol-gel fabrication pathway of different ZnO nanostructures from changing environments to produce their variable morphologies from a single source. The application of these nanostructures for quantitative interaction with insulin in aqueous solution, would offer ample opportunities for quantitative determination of biomolecules with other nanoparticles.

## 2 Materials and methods

### 2.1 Materials

Purified zinc nitrate hexahydrate was procured from Merck Limited, Mumbai-400018, and used as the source of Zn. The milk is collected from the local market as fresh liquid milk. Insulin (human) R 100 IU/mL was purchased from a local market. Sodium hydroxide (NaOH) was collected from Active Fine Chemicals Limited, Dhaka, Bangladesh. Ethanol was purchased from Merck KGaA, 64,221 Darmstadt, Germany. Demineralized water was produced using the equipment WDA300.RW1.5 (Fistreem International Ltd., UK). Distilled water was obtained using the equipment WCA004.MH1.4 (Fistreem International Ltd., UK).

Here, ZnO NPs were synthesized using the eco-friendly Sol Gel process. No adverse solvent is used in the whole synthesis process. From the literature survey, it is known that naturally occurring carbohydrate molecules have the ability to provide a sufficient reaction site or work as substrates for the synthesis. Here, cow's milk was used as a solvent. About 5% of milk content is lactose, which is a diasaccharide (galactose and glucose). Zn(NO_3_)_2_.6H_2_O worked as a source of zinc. To make the solution at desired pH, NaOH was added. Demineralized water was added at the time of the reaction. The fresh cow's milk was stored in the refrigerator at −18 °C.

### 2.2 Synthesis of ZnO NPs

About 75 mL of thawed cow milk was heated at 65 °C using a hot plate (hot plate stirrer LMS-1003, Daihan Labtech Co., Ltd., Korea). About 7.5 g of Zn(NO_3_)_2_.6H_2_O were dissolved in 50 mL of demineralized water and stirred for 10 min to form an almost clear solution. The pH value (microprocessor pH meter, pH 211 HANNA Instrument) of the Zn(NO_3_)_2_.6H_2_O solution was measured to be ca. 2. This acidic solution was added to the milk while the whole solution was stirred with a magnetic stirrer. As the pH of the milk declined, the protein portion get separated and screened from the solution. In this environment, the solution took on a pale green color. Then the variable quantities of 0.5 M NaOH solution were added separately to fix the pH of the solution from pH 6 to pH 10. After that the reaction was conducted for 30 min at 65 °C, and the whole solution turned into a white, dense liquid. Later, the solution was cooled and centrifuged (D-78532, Hettich) at a speed of 6000 rpm for 10 min to isolate the solid. Then the solid was dried in an atmospheric dryer at 65 °C and calcined at 500 °C for about 2 h. The yield of the reaction was determined from the clear liquids left by centrifugation, which were subjected to gravimetric analysis to quantify the amount of zinc present in the liquid.

### 2.3 Characterization of ZnO NPs

The conformational wavelength of zinc nanoparticles in ethanol was determined using a double-beam UV-1700 Series UV-VIS spectrophotometer. FT-IR spectra were recorded on a FT-IR 8400S spectrophotometer (Shimadzu Corporation, Japan) in the wave number range of 4000-400 cm^−1^, resolution: 4 cm^−1^, scan: 30. XRD patterns were recorded on an x-ray diffractometer (ultima IV, Rigaku Corporation, Japan using Cu K_α_ radiation (λ = 0.154 nm, 40 kV, 40 mA). The scan speed and scan range were 3°/min and 10–70°. The morphology of the sample was recorded using a scanning electron microscope (JEOL JSM-6490LA, Tokyo, Japan). The operating voltage was 20 kV and secondary electron imaging mode was used for image collection. For this study, the sample was dispersed in ethanol and sonicated for 10 min to disintegrate the agglomerated particles. The dispersed solution was then spread on a glass slide and allowed to dry by evaporation of ethanol at atmospheric conditions. The hydrodynamic diameter of the samples was measured using a Zetasizer Nano ZS90 (ZEN3690, Malvern Instruments Ltd., UK) by the dynamic light scattering method (DLS). A He–Ne laser of 632.8 nm wavelength was used as a light source. The elemental composition of ZnO was determined by recording the EDS spectra on EDAX elemental analyzer.

## 3 Results and discussion

### 3.1 Effect of pH on ZnO NP formation

**Fig. 1:**
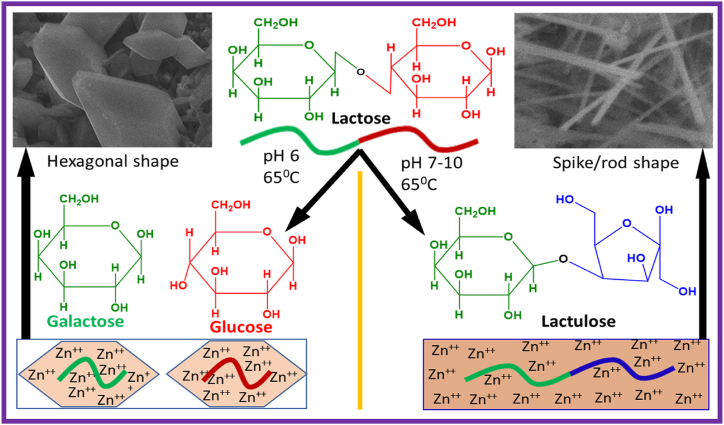
Schematic presentation of lactose-assisted and pH-regulated ZnO NPs synthesis.

Nanostructured ZnO particles with different morphologies were synthesized via an eco-friendly lactose-assisted pH-regulated sol-gel method. The nanostructured ZnO particles synthesized at pH 6, 7, 8, 9, and 10 are hereafter denoted as pH 6, pH 7, pH 8, pH 9, and pH 10. As the pH of the solutions varied from 6 to 10, the zeta potential also changed. These twisting environments lead to formation of different species from lactose as shown in Fig. 1. Therefore, the interaction between lactose and its species with zinc cations varies with different assembly. The rate of migration of cations for construction of crystal morphologies may also determine the size of nanostructure in the solution [30]. Fig. 1 shows that at 65 °C, a pH 6 (slightly acidic) solution produced a hexagonal-shaped crystal structure, but the pH from 7 to 10 fabricated different sizes of rod-shaped or spike-like nanostructures.

It has previously been reported that nanoparticles readily formed aggregates and could not be disintegrated to their original size despite the application of ultrasonication. The stability of ZnO suspension as a function of time has changed in zeta potential of its suspension as a function of pH. Subsequently, the effects of pH on zeta potential and hydrodynamic diameter as a function of time are also changed for ZnO nanoparticles unique character [40].

**Fig. 2:**
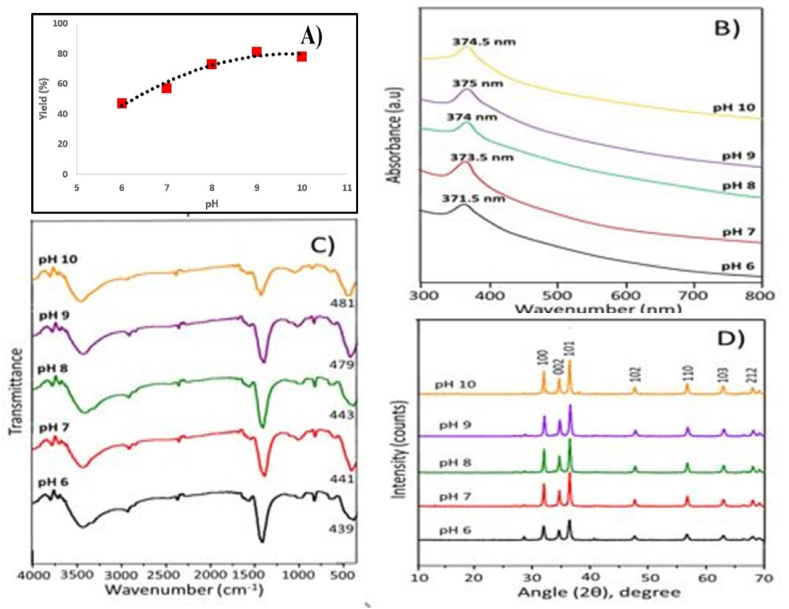
Plots for (A) percentage yield of ZnO nanoparticles with variation of pH of reaction solution, (B) UV–Vis spectra, (C) FT-IR spectra, and (D) XRD patterns of ZnO NPs produced at pH range 6 to 10.

In Fig. 2A, the yield of the synthesized nanostructure vs. pH is plotted. About 45% yields were obtained from pH 6 solutions, but the maximum yield was found from pH 9 solutions. It is evident that the yield of nanostructured ZnO particles increased with the increase in pH up to 9. At pH 10, the yield was slightly lower than that of pH 9, which may be due to the analytical error or the conversion factor involved with the specific pH. The variation of yield depending on pH changes due to the formation of micelles number, quantity and their sizes in solution.

### 3.2 UV–vis analysis

UV–visible spectral analyses were carried out for all synthesized particles and plotted in Fig. 2B. The spectra revealed a characteristic absorption of ZnO at wavelengths between 371 and 375 nm, which can be attributed to the intrinsic band-gap absorption of ZnO due to the electron transition from the valence band to the conduction band [[3], [4], [5], [6],^12^,^13^,^17^]. With a change in pH, the shift in the absorption peak indicates a change in particle size. Calcinated (at 500 °C) ZnO particles produced at different pHs show different maxima due to their different sizes.

It is envisaged that with the increase in pH, the maxima will shift towards red, which indicates the increment of particle size in solution. Although compared to the UV–visible absorption spectrum of bulk ZnO NPs (382 nm), it is blue-shifted (ca. 10 nm) (Fig. 2B, pH 6) [5].

The absorption band exhibited an unusual blue shift, which can be explained by the presence of a very thin bridge between the produced nanoparticles. Absorption at the wavelength of 373.5 nm confirms that the ZnO NPs' absorption spectrum is slightly blue-shifted relative to its bulk value (382 nm) due to the nanostructures' size effect [5] and also confirms the quantum confinement effect among the individual nanoparticles (Fig. 2B, pH 7) [[3], [4], [5], [6],^12^,^13^,^17^]. Haiping et al. explained the fact that the particle size-dependent visible spectrum varies with ZnO NPs size because the visible emission center is created due to the trapping of photogenerated holes in the surface and the tunneling back of the electron to recombine with oxygen [41]. A similar effect was found for the nanostructured ZnO particles synthesized at pH 8, 9, and 10.

### 3.3 FTIR analysis

Functional groups of synthesized nanostructured ZnO particles were determined using FT-IR spectral analysis. Fig. 2C shows the FT-IR spectra after calcination at 500 °C of particles produced from pH 6, pH 7, pH 8, pH 9, and pH 10, and in all cases the presence of ZnO was confirmed with strong peaks at 439, 441, 443, 479, and 481 cm^−1^, respectively. The region between 400 and 600 cm^−1^ is attributed to the Zn–O bond. The change in characteristic peak position may be due to variations in particle size, shape, or crystallinity. In our previous article, we showed that cow milk contains some cations of K, Ca, Mn, Fe, and Cu in addition to Zn, which may also form their oxides at elevated temperatures and gave peaks of 651, 680, and 702 cm^−1^. The peaks at 1149 and 1155 cm^−1^ are attributed to the C–O bond stretching of C–*O*–C and C–*O*–H groups in lactose, respectively, used as dispersing and reducing agents during the fabrication of ZnO nanoparticles [5]. It is illustrated from Fig. 2C that as the pH increases, there is a little shift of the peak at lower frequencies, and that peak shift is from 439 to 481 cm^−1^ for pH increases from 7 to 10. The broad peak in the higher energy region at 3000–3700 cm^−1^ is due to the stretching vibration of the OH group [5].

### 3.4 XRD analysis

The XRD patterns (Fig. 2D) recorded from ZnO NPs showed peaks at 2θ values at 31.8, 34.45, 36.28, 47.6, 56.57, 62.82, 67.96, and 69.19° indexed to the (100), (002), (101), (102), (110), (103), (212), and (201) planes, respectively. Those peaks are completely matched with the reference JCPDS card no. 36–145, which confirms the formation of ZnO nanoparticles from Zn(NO_3_)_2_.6H_2_O using lactose from cow's milk [42]. There are some other peaks at 2θ values of 26.84 and 28.46° that resemble ZnO nanoparticles and may be incorporated from the elements present in the milk sample. The crystallite sizes of ZnO nanoparticles at pH 6, 7, 8, 9, and 10 were calculated to be 23.23, 20.41, 30.02, 28.46, 25.40, and 28.64 nm, respectively [43]. It is noteworthy to mention that solution pH also regulates the size of ZnO. It has been found that the XRD spectrum of ZnO nanoparticles fabricated from pH 7 showed sharper peaks and produced a 30.02 nm crystallite size, although the average crystal size of the nanoparticle was found to be 26.02 nm.

### 3.5 Elemental analysis

**Fig. 3:**
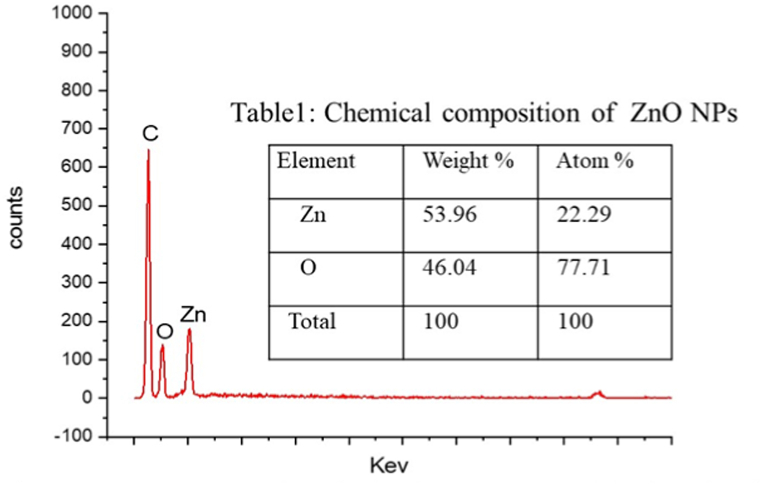
EDX spectrum of synthesized Zno nanoparticle from batch pH 9.

The EDX analysis (Fig. 3, pH 9) confirmed the chemical composition of the synthesized ZnO NPs. The strong and weak peaks are observed in the Zn and O atoms. About 54 and 46 wt% of zinc and oxygen were present in the ZnO nanoparticles (Table 1), which indicates some foreign elements like carbon and other oxides of minerals from milk contributed to the formation of oxygenated compounds [43,44].

### 3.6 DLS analysis

**Fig. 4:**
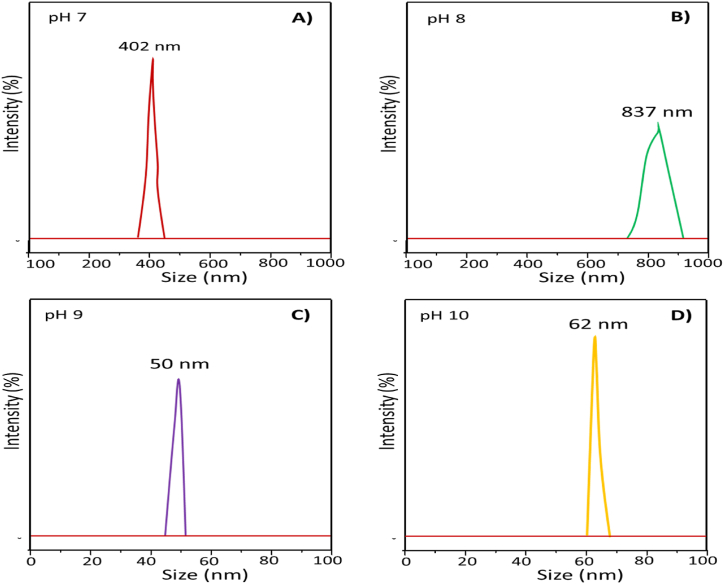
Determination of hydrodynamic size of ZnO NPS fabricated from pH (A) 7, (B) 8, (C) 9 and (D) 10 by DLS technique using 1 mg/mL in ethanol.

Fig. 4(A-D) shows the trace of DLS for ZnO nanoparticles fabricated from pH 7 to 10 using 1 mg/mL in ethanol. It is revealed from DLS data that the hydrodynamic diameter of ZnO NPs were measured to be ca. 402 nm, 837 nm, 50 nm, and 62 nm for pH 7(Fig. 4A), pH 8 (Fig. 4B), pH 9 (Fig. 4C), and pH 10(Fig. 4D), respectively. The smallest size (50 nm) was fabricated at pH 9, and the biggest size (837 nm) was formed at pH 8. Whereas, in a neutral environment, pH 7 produced a moderate size of ZnO nanoparticles, which was almost half (402 nm) of the particle size produced at pH 8 (837 nm). Interestingly, with increasing alkalinity (pH 9–10), the particle size became smaller (50–62 nm) [42]. In an acidic solution at pH 6, a hexagonal-shaped particle was formed. This particle did not persist in ethanol long enough to determine its size. It indicates that the ionic strength of the solution would determine the shape and size of the particles. The variation in size could be due to the ionic interaction between the lactose species and zinc cation and the rate of accumulation and simultaneous reduction of zinc cation onto the surface of the dispersing agent. Besides, DLS measures the particle size in the solvated state; therefore, the agglomeration and stability of particle dispersions depend on the attractive and repulsive forces between individual particles as well as the thixotropic effect. The average hydrodynamic diameter increased dramatically with increasing solution ionic strength [45,46]. Based on the size distribution and morphology, efficacy of the ZnO nanoparticles determines the field of applications [2,3,5,12,13,17,23,38,39].

### 3.7 SEM analysis

**Fig. 5:**
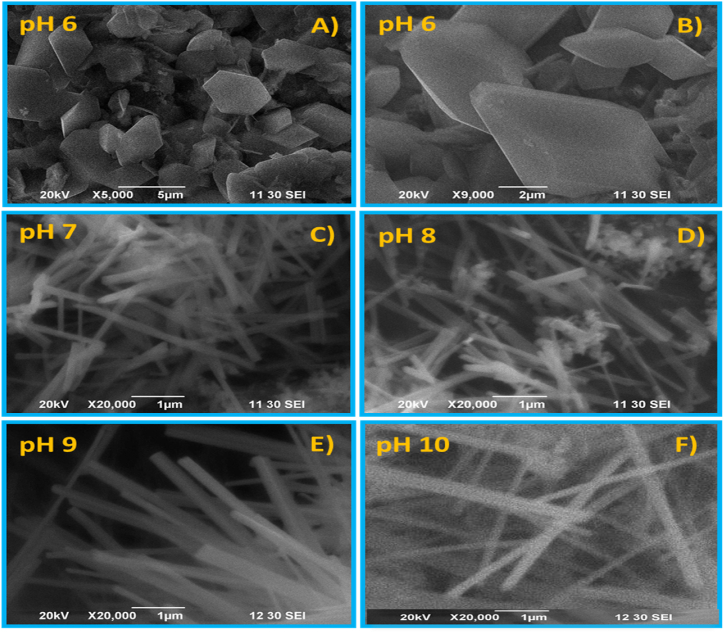
SEM images of pH-regulated lactose-inspired ZnO NPs at (A,B) pH 6, (C) pH 7, (D) pH 8, (E) pH 9, and (F) pH 10.

The SEM images show hexagonal, rod, and some other spike-like particles [[3], [4], [5]]. Fig. 5 shows the morphologies ZnO NPs synthesized at different pH. It is illustrated in Fig. 5A,B that at acidic pH 6, a hexagonal shape with 4–5 μm-sized particles was formed. The structure was turned into a rod shape with about 3 μm length and 150 nm width when pH was changed to neutral (pH 7), as shown in Fig. 5C. Fig. 5D represents an image of ZnO nanoparticles with a mixture of rod and strip-like structures produced at pH 8. Whereas Fig. 5E, F shows images obtained at pH 9 and 10, in these conditions the particle morphology remained almost unchanged, although rod-like particles with a very sharp edge of flat surface about 250 nm width were found at pH 9. But the particle morphology is sharply tuned to a round rod structure for pH 10. It can be stated that the morphology of ZnO NPs was regulated by a pH-controlled reaction environment, which is reflected in SEM images with different sizes and shapes of particles.

### 3.8 Sensing of insulin with ZnO NPs in an aqueous system

Lactose-assisted ZnO NPs were used to sense the biomolecule insulin in the aqueous system. Since the synthesized ZnO NPs sizes and shapes were different, the interaction between insulin and particles was investigated using all types of ZnO NPs fabricated at pH 7–10. The pH-6-controlled ZnO particles were bigger and were not retained in an aqueous environment. Due to their gravity, these ZnO NPs limit their application for insulin sensing. A constant concentration (25 μM) of ZnO NPs was used to carry out UV–Vis spectra acquisition with varying concentrations of insulin ranging from 2.5 to 50 μM. The maximum absorbance (a.u.) of UV–vis spectra generated from LSPR behavior of all ZnO NPs was recorded for sensing with respect to insulin concentration [41].

**Fig. 6:**
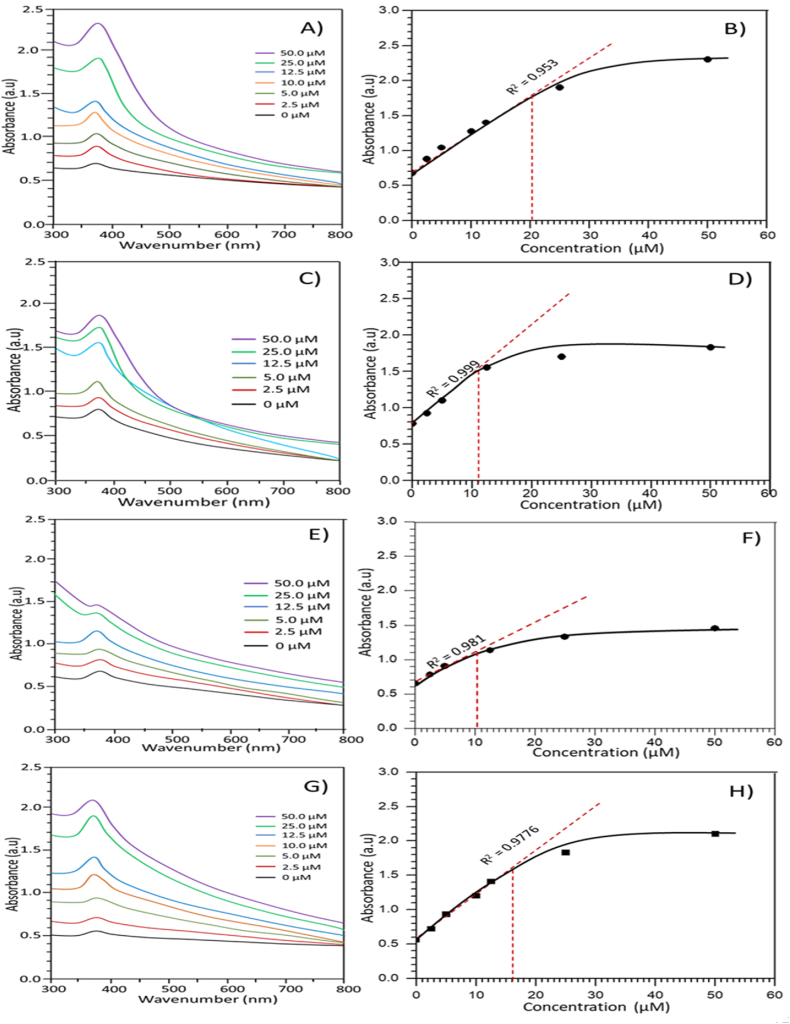
UV–Vis spectra and absorbance vs. insulin concentration (μM) plot for ZnO NPs fabricated from (A,B) pH 7, (C,D) pH 8, (E,F) pH 9, and (G,H) pH 10.

Fig. 6 shows all UV–vis spectra recorded for different ZnO NPs with insulin concentrations and the corresponding plot from UV–vis spectral absorbance (a.u.) vs. insulin concentrations. In Fig. 6A, B, UV–Vis spectra and corresponding plots for ZnO NPs fabricated from pH 7 are presented. The bare ZnO NPs at 25 μM in ethanol gave a maximum absorbance of 0.68 at 374 nm [5]. When 2.5 μM insulin was added to the ZnO NPs solution, the peak position was almost the same, but absorbance increased to 0.88. Likewise, absorbance increased linearly with increasing insulin concentrations up to about 25 μM (absorbance 1.9) with a regression value of R^2^ = 0.953. It indicates that the addition of insulin changed the microenvironment in the vicinity of ZnO NPs, leading to an enhancement of absorbance. The linearity of the curve in Fig. 6B indicates the susceptibility of the ZnO NPs for insulin sensing. At higher concentrations, i.e., more than 20 μM, the line deflected from the curve, probably due to the solution's overall turbidity. Interestingly, at high concentrations, the absorption peak was very sharp to detect, which suggested that if the solution became transparent, the linearity of absorbance would be increased. Similarly, lactose-inspired ZnO NPs from pH 8, 9, and 10 were used to sense insulin. Fig. 6 (C, D); (E, F); and (G, H) shows UV–Vis spectra and corresponding plots. The bear particles showed very clear maximum absorbance peaks at 0.78, 0.65, and 0.56 a.u. for ZnO NPs fabricated from pH 8, 9, and 10, respectively. The UV–vis spectra recorded for ZnO NPs at pH 9 in the presence of higher insulin concentrations were very weak. This could be due to an unusual environment arising in the solution from excessive or less interaction with insulin. It is evident that the linearity of absorbance was very low for ZnO NPs from pH 8 and 9 up to about 12 μM, but a good linearity was also found for ZnO NPs synthesized from pH 10 up to about 16 μM of insulin with a regression value of 0.9776 as shown in Fig. 6 (G,H) [35,38,39]. Table 1 summarizes the insulin sensing performance of ZnO NPs at pH 7 and 10.

**Table 1:** Important parameters involved in insulin sensing by ZnO NPs at pH 7 and 10.

Parameters	At pH 7	At pH 10
Regression	0.953	0.978
Limit of detection	>1 μM	> 1 μM
Limit of quantification	1–20 μM	1–16 μM
% Yield	67.5	79.4
*λ*_max_	373.5 nm	374.5 nm

### 3.9 Mechanism of insulin sensing by ZnO NPs

**Fig. 7:**
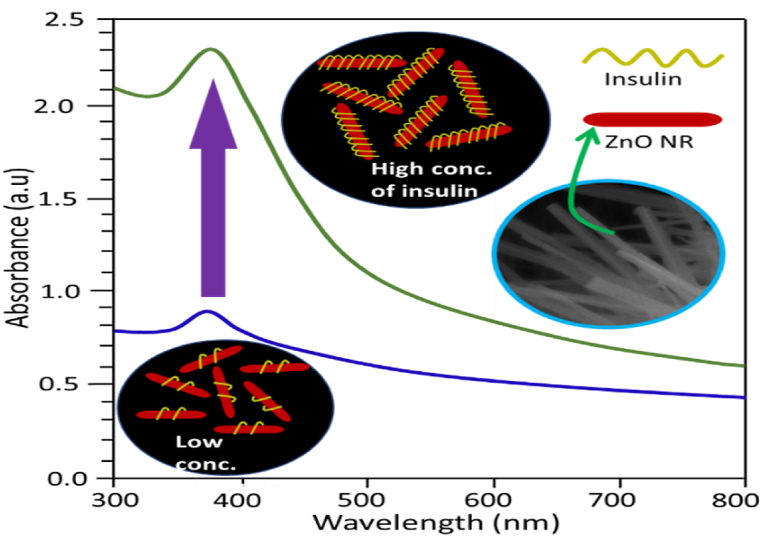
Mechanism involved for sensing insulin by ZnO NPs.

Insulin biomolecule sensing mechanisms are directly involved with the LSPR behavior of lactose-assisted pH-regulated ZnO NPs. The LSPR behavior of a particular particle depends on its size, shape, and morphological structure [[36], [37], [38]]. Fig. 7 demonstrates the possible mechanism involved in the sensing of insulin by ZnO NPs in ethanol. At low concentrations of insulin, the interaction between insulin and ZnO NPs was low, hence the low absorption. Interaction between ZnO NPs and insulin depends on the solution environment, solute physical and chemical properties, and substrate surface phenomena. The rod-shaped ZnO NPs showed an absorption peak due to their inherent LSPR characteristic at around 374 nm. This absorption peak was intensified with the interaction of insulin. Because insulin provided a new environment around the ZnO NPs, which accelerated the absorption capacity of light at certain wavelengths created by LSPR, Therefore, the wrapping of ZnO NPs by soft insulin biomaterials up to a certain level of concentration maintains consistency.

Rod-shaped 402 nm ZnO NPs fabricated at pH 7 gave a harmonic UV–visible absorbance, but 837 nm ZnO NPs fabricated at pH 8 showed low insulin sensing ability due to the size effect. Whereas, 50-nm ZnO NPs fabricated from pH 9 showed an abnormal UV–visible spectral pattern. The SEM image showed a ribbon-like structure that could be less interacted with by insulin and perturbed by its environment. Besides, 62 nm ZnO NPs fabricated from pH 10 with a rod-shaped structure produced a smooth UV–visible spectral pattern, which resembled the results of ZnO NPs fabricated from pH 7 [35,38,39].

## 4 Conclusion

This study reports the LSPR dependent optical sensing of insulin by ZnO nanoparticles. An eco-friendly green method has been utilized in this study to fabricate ZnO from zinc nitrate precursor at different pH using lactose as a reducing agent. A key finding of this study is that the pH of the reaction media strongly controlled the morphology of the ZnO NPs. Acidic media (pH 6) resulted in the formation of hexagonal ZnO NPs while rod-shaped nanoparticles were formed in the alkaline media (pH 7–10). In addition to the shape, the size of ZnO NPs was also controlled by pH. The size and shape of ZnO strongly influences its optical and electrochemical sensing ability of analyte molecules. ZnO nanorods synthesized at pH 7 demonstrated the best LSPR sensing of insulin within a linear concentration range of 2.5–20 μM with high confidence. The morpholigcal advantage of rod-shaped ZnO nanoparticle is that it has a high surface area that helps making a better interaction with insulin than other shapes. The potential application area of insulin sensing by ZnO nanorods will be real time monitoring of glucose for efficient management of diabetes. The future research should consider the modification of the ZnO nanorods via chemical functionalization with antibodies or receptors for improved sensing performance.

## Author contribution statement

Nasim Ahmed: Performed the experiments; Wrote the paper.

Shaikat Chandra Dey: Analyzed and interpreted the data; Wrote the paper.

Nusrat Mustary: Analyzed and interpreted the data; Wrote the paper.

Md. Ashaduzzaman: Conceived and designed the experiments; Contributed reagents, materials, analysis tools or data; Wrote the paper.

## Data availability statement

Data will be made available on request

## Additional information

No additional information is available for this paper.

## Declaration of competing interest

The authors declare the following financial interests/personal relationships which may be considered as potential competing interests:
